# Repair Bond Strength of Aged Resin Composite after Different Surface and Bonding Treatments

**DOI:** 10.3390/ma9070547

**Published:** 2016-07-07

**Authors:** Michael Wendler, Renan Belli, Reinhard Panzer, Daniel Skibbe, Anselm Petschelt, Ulrich Lohbauer

**Affiliations:** 1 Dental Clinic 1-Operative Dentistry and Periodontology, Research Laboratory for Dental Biomaterials, Friedrich-Alexander University Erlangen-Nuremberg, Erlangen 91054, Germany; mwendler@dent.uni-erlangen.de (M.W.); renan.belli@dent.uni-erlangen.de (R.B.); reinhard.panzer@gmx.de (R.P.); dskibbe_88@yahoo.de (D.S.); anselm.petschelt@dent.uni-erlangen.de (A.P.); 2 Department of Restorative Dentistry, Faculty of Dentistry, University of Concepción, Concepción 4070369, Chile

**Keywords:** aged resin composite, bonding agent, resin composite repair, surface treatment, tensile bond strength

## Abstract

The aim of this study was to compare the effect of different mechanical surface treatments and chemical bonding protocols on the tensile bond strength (TBS) of aged composite. Bar specimens were produced using a nanohybrid resin composite and aged in distilled water for 30 days. Different surface treatments (diamond bur, phosphoric acid, silane, and sandblasting with Al_2_O_3_ or CoJet Sand), as well as bonding protocols (Primer/Adhesive) were used prior to application of the repair composite. TBS of the specimens was measured and the results were analyzed using analysis of variance (ANOVA) and the Student–Newman–Keuls test (α = 0.05). Mechanically treated surfaces were characterized under SEM and by profilometry. The effect of water aging on the degree of conversion was measured by means of FTIR-ATR spectroscopy. An important increase in the degree of conversion was observed after aging. No significant differences in TBS were observed among the mechanical surface treatments, despite variations in surface roughness profiles. Phosphoric acid etching significantly improved repair bond strength values. The cohesive TBS of the material was only reached using resin bonding agents. Application of an intermediate bonding system plays a key role in achieving reliable repair bond strengths, whereas the kind of mechanical surface treatment appears to play a secondary role.

## 1. Introduction

One of the most important precepts in modern dentistry is minimally invasive intervention. Dentists are challenged to avoid unnecessary damage to sound dental tissues and to limit removal to what is strictly necessary. Nevertheless, more than half of all repairing procedures still consist in total replacement of the defective restorations [1], with a consequent higher sacrifice of the healthy tooth [2,3]. Different clinical studies have demonstrated that repair, refurbishing or sealing damaged resin composites are reliable alternatives for replacement [4,5,6], effectively improving the longevity of the restorations [2,3,7]. On the other side, great efforts have been undertaken in the last two decades to understand and improve the repair process, given the unpredictable results observed by bonding to aged or contaminated resin composites [8]. In contrast to other chair side repair procedures (i.e., following ceramic chippings), no broadly accepted protocol for resin composite repair has been established yet.

The joint between old and new resin composite has been described to occur by three possible mechanisms: (I) micromechanical retention by penetration of the new monomers into the irregularities of the treated surface; through chemical bonding of these monomers; (II) to the matrix; and/or (III) to the exposed filler particles [9]. Mechanical surface treatment is achieved by grinding with diamond burs or by sandblasting the surface with aluminum oxide (Al_2_O_3_) particles, creating a micro-retentive surface that enables mechanical interlocking of the new material [10,11,12]. Additionally, removal of the superficial layer, chemically altered by the long exposition to the oral environment, enhances the surface energy of the old resin composite [13,14]. Sandblasting with silica coated particles instead of pure Al_2_O_3_ has shown to add an additional chemical bonding [15,16] by incrementing the silica content in the surface, especially if a silane coupling agent is used prior to application of an adhesive system [13,15,17]. On the other hand, the use of a low viscosity bonding agent has proven to increase repair bond strengths [18,19,20,21] by deeper infiltration into the micro-retentions created by the surface treatment [11], but also by direct chemical interaction with the unconverted C=C double bonds on the aged resin composite surface [14]. The rate of copolymerization of the new material with these unreacted groups has been claimed to be determinant for the repair bond strength [22].

Quantification of the bond strengths between the old and the new material has been extensively used in the literature as a success parameter of the repair process. Although in vitro testing conditions do not truly represent the complexity of the oral environment, they allow comparison of the effects of different repairing protocols under controlled conditions. Complementary characterization of the treated surface along with fractographic assessment of the fractured interfaces delivers useful information to understand the repair bonding process. Therefore, the aim of the present study was to compare the effect on the tensile bond strength (TBS) of different mechanical surface treatments and bonding protocols on aged resin composite. In addition, the treated surfaces were characterized by means of profilometry and SEM. The effect of water aging on the resin composite degree of conversion (DC) was assessed via FTIR-ATR spectroscopy.

## 2. Results

**Figure 1:**
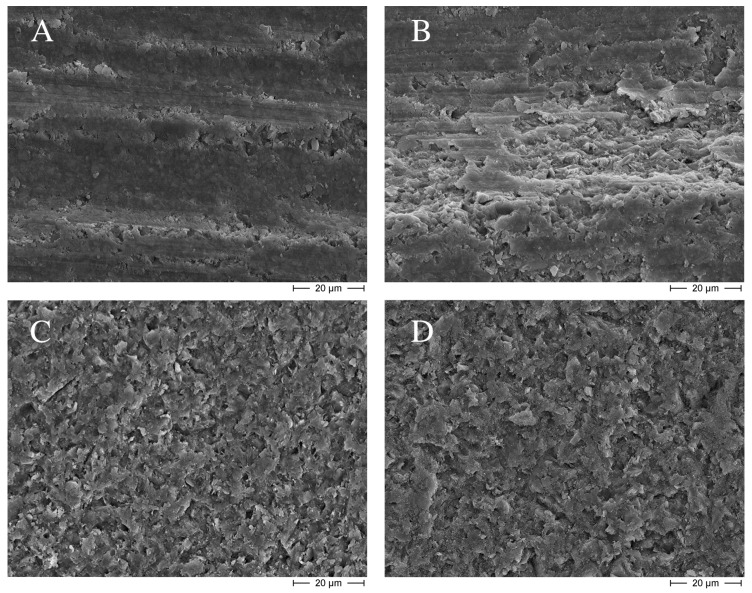
Scanning electron micrographs of the different surface treatments: (**A**) Negative reference, red code diamond bur; (**B**) Group 3, blue code diamond bur; (**C**) Group 4, sandblasting with Al_2_O_3_ particles; and (**D**) Group 6, sandblasting with CoJet Sand. Etching with phosphoric acid (Group 2) did not affect the microscopic appearance of the surfaces, which looked similar to those of the negative reference (**A**) and were therefore not presented here.

Table 1 presents the TBS results. No statistical differences were observed among groups with different surface treatments (2 to 6), although bond strengths were significantly higher than the negative reference. This increase in TBS, however, was not correlated to the profilometric results of the treated surfaces (Table 2). As expected, the blue code bur created the roughest surface, while both sandblasting procedures slightly smoothened the surface generated by the red code bur. SEM observations (Figure 1) corroborated the effect of sandblasting in generating smoother surfaces, which were also more homogeneous that those created by the diamond burs. Silane application with a previous surface silicatization (Group 6) did not show statistically higher TBS values than the sole application of silane (Group 5).

The application of the bonding system (Groups 7 to 11) significantly improved the TBS values, reaching the bond strength of the positive reference groups. The best results were obtained with the application of the complete sequence of Syntac and Heliobond after phosphoric acid etching (Group 11), although its TBS values were no statistically different from those of Groups 7 to 10. No differences were observed in TBS between the positive references after water storage.

The failure mode was adhesive for all experimental groups, as well as for the negative reference. Application of the complete bonding system (Group 11) led to a 20% of mixed fractures, whereas only few specimens in Groups 9 and 10 had this failure mode. The positive references showed only cohesive fractures.

Results of the FTIR-ATR spectroscopy showed a higher degree of conversion after water storage (70.4% ± 1.5%) than for the non-stored specimens (58.7% ± 5.1%), pointing out a decreased amount of unreacted double bonds after aging.

## 3. Discussion

The ultimate goal of repairing a resin composite restoration is to achieve durable bond strengths between old and new material, ideally matching the inherent strength [8]. Therefore, in the present study, positive reference groups were included, representing the maximal repair potential in relation with its cohesive strength [23]. This was confirmed by the fractographic analysis, as all specimens in these groups failed cohesively. The negative reference, on the other side, served as baseline for all experimental groups, and consistently obtained the lowest TBS values. Indeed, all experimental groups ranged between both references. In order to isolate the effect of the mechanical treatment on the bond strength, Groups 1 to 4 did not receive any further chemical treatment. Although this does not correspond well with clinical procedures, where adhesive systems are commonly applied, it allowed a better appreciation of the influence of surface texture on the bond strength. Similarly, the separate application of the adhesive system’s components on specimens having only a baseline mechanical treatment (Groups 7 to 9) served the purpose of determining their individual contribution to the final bond strength.

Bonding of resin composite layers is achieved primarily by covalent chemical bonds [24] between unreacted groups of the first cured layer and the monomers of the newly applied one, being also favored by the lower viscosity of this partially polymerized first layer [25]. Therefore, any condition decreasing its reactivity will lead to reduced interfacial bond strengths [24]. In the present study, positive control groups, where an inhibition layer was permitted to form, showed no interfacial failures, confirming its contribution to the bonding process. On the other hand, the importance of this unreacted layer for resin composite repair has been questioned in recent years, since no positive effects have been observed on the repair bond strengths values [17,20,26]. Additionally, most clinical repairing protocols include surface roughening, which causes removal of this superficial layer before the new material is applied. Accordingly, the material removal of 0.3 mm accomplished here on the specimens’ surfaces after aging aimed to mimic these clinical procedures.

As stated before, the presence of unreacted C=C double bonds in the treated surface layer plays a critical role for the adhesion of the new material [22,27]. Availability of these unreacted groups depends principally upon chemical composition of the matrix and the aging history. The nano-hybrid methacrylate based resin composite used in the present study combines three different monomers that account for its chemical properties. Whereas the large bis-GMA (Bisphenol A diglycidylmethacrylate) molecules have higher viscosity and reduced DC, the smaller and flexible TEGDMA (triethylene glycol dimethacrylate) is used as diluting monomer, increasing the DC [28]. Additionally, the modified monomer bis-EMA (Ethoxylated bisphenol A dimethacrylate) has shown to increase the DC and decrease water sorption [29]. A mean 58.7% DC was measured 16 h after light curing in this study, which is within the range of values measured for this material in the literature [30,31]. An important increase in the DC was observed after 30 days water storage for the samples, with a mean value of 70.4%. Water sorption is a diffusion-controlled process, dependent upon hydrophilicity of the constituent monomers [32,33], causing leaching of unreacted monomers, swelling and degradation of the matrix-filler interface [34,35]. Additionally, water saturation of the resin composite has been claimed to reduce the available free radicals and thus decrease repair bond strengths [8,14]. Despite the reduction in double bonds observed after water storage of the samples, the obtained DC value corresponded well with those informed for the same material after 24 h dry storage [30,31]. It seems that the maximal DC was reached in the first 24 h, and no further increase occurred, regardless of the applied storage conditions.

Micromechanical retention on the aged surface has been reported as one of the key mechanisms to achieve reliable repair bond strength [9,10,11,36,37]. Nevertheless, no consistent correlation has been established between the roughness profile of the treated surfaces and the bond strengths achieved [18,38]. In the present study, the use of a blue code bur generated higher surface roughness (Ra of 3.36 µm) than the red code bur (Ra of 1.07 µm), and led to higher bond strengths. Contrarily, da Costa et al. [38] found no significant differences in tensile bond strength values among surfaces treated with varying diamond bur grits, suggesting that even if roughness profiles were different, micro-retention patterns were similar. On the other hand, roughness profiles of the sandblasted groups were smoother than those of the red code bur (Table 2), but the bond strengths achieved after these treatments were significantly higher (Table 1). Surfaces treated with diamond burs appear to have more macro-retentive features [9,27], being more irregular [14] and barely micro-retentive (Figure 1A,B), while sandblasting creates more homogeneous surfaces (Figure 1C,D), with dominating micro-retentive features [9,27,39]. Accordingly, the total adhesion area produced by sandblasting would be higher than that generated by diamond burs, despite eventual similarities in Ra [14]. However, bond strengths seem not exclusively rely on micro-retentive features if no underlying resin bonding agent is used [9,19], as observed for the similar TBS values between sandblasting groups and the blue code bur. Moreover, when a bonding agent was applied on the previously bur-roughened specimens (Groups 7–11), TBS results were significantly higher than those of sandblasted surfaces.

No significant differences were observed among the sandblasting groups, neither in TBS values, nor in the surface roughness profiles. This is in agreement with other published studies [14,23]. Although interaction between the silica modified particles of CoJet Sand embedded in the resin composite surface and the resin matrix of the repair material is expected to enhance bond strengths, especially after subsequent silane application [15,16], no such contribution was observed in the present study. The comparable mean size of the abrasion particles (30–35 µm) accounted for the similar SEM pictures (Figure 1C,D). They resembled well SEM examinations by Rathke et al. [18], which also observed no incorporation of abrasion particles into resin composite surfaces. This can lead to the assumption that the enhanced bond strengths by the sandblasting procedures were solely due to the micro-retentions created on the surfaces [40], as discussed before.

Application of silane on the silica coated surface (Group 6) slightly enhanced the bond strength compared to the solely silanized surface (Group 5), although the difference was not statistically significant. Both groups had higher bond strengths than the negative reference, even though they were significantly lower than the positive controls. The silane coupling agent forms covalent bonds with the exposed filler particles in the aged resin composite surface and co-polymerizes with the methacrylate groups of the repair material [16,41], enhancing the repair bond strength. This effect is particularly relevant on aged resin composites, where degradation and loss of the silane layer of the inorganic fillers is expected [15,24]. Additionally, silane improves the wettability of the surface, facilitating diffusion of the bonding agent into the micro-retentions in the substrate [15,17,23]. Although many studies have described higher repair bond strengths after silane application [8,40,42,43,44], especially following sandblasting with silica particles [13,15,17], some others did not observe any significant improvement in the bond strength [9,10,18,19,38,45]. Depending on the amount of filler available at the surface, its nature [43] and size [42], silane does play a key role in enhancing repair bond strengths. Still, this remains a controversial issue.

Etching with phosphoric acid is a routine step in resin composite repair procedures, as normally adjacent enamel and/or dentin have to be conditioned to achieve adequate bond strengths. Nevertheless, most studies have limited its contribution in the composite–composite bonding process to the removal of debris and grinding dust [13,20,23,27]. Although its application has shown to increase the total surface area [44], no improvements in bond strength have been observed [15,19], leading to clinically unacceptable repair bond strengths [44,46]. Accordingly, in the present study application of phosphoric acid had no effect on the roughness of the treated surface and the TBS values did not match the positive reference groups. Even though, a significant increase in bond strength was observed with respect to the negative reference, achieving equivalent values to those of the other surface treatments (Table 1). Elimination of surface debris and filler exposure [44] enhanced surface energy and wettability of the surface, promoting adhesion to the aged material. Moreover, subsequent application of the bonding system (Group 11) produced the highest repair bond strengths in this study. Loomans et al. [23] obtained similar results and thus recommended this protocol for clinical repair of aged resin composites.

Application of a bonding system yielded the best TBS results, even reaching the cohesive strength of the material (Table 1). The positive effect of bonding agents on the bond strength is strongly related with the limited penetration capacity of the repair resin composite material into the surface microstructure, due to its high viscosity [44,47]. Additionally, a reduced chemical potential in the substrate is expected after the aging process [26,47]. Intermediate unfilled resins enhance chemical bond to the matrix and to the exposed fillers [9,21], as well as improve micromechanical retention by infiltrating into the micro-irregularities created by the mechanical treatment on the surface [11]. Furthermore, a non-polymerized layer is created on the aged surface by oxygen inhibition, which may aid adhesion of the new material [15,42]. Syntac is a multiple-component adhesive system, that includes both hydrophilic (Primer and Adhesive) and hydrophobic (Heliobond) unfilled resins. Separated (Groups 7–9) as well as combined application of the components (Group 10) had no significant effect on the TBS achieved. Similar results were observed by Rathke et al. [18] for another adhesive system, with no differences in bond strength between hydrophilic and hydrophobic resins. Therefore, utilization of additional hydrophilic primers could be limited to clinical situations where dentin is also involved in the repair procedure [18]. Alternatively, Papacchini et al. [48] proposed the utilization of flowable resin composites as intermediate material, principally owing to their superior mechanical properties and their higher stress-absorbing ability. However, beyond differences in filler content and/or hydrophilic properties, their low viscosity and high wetting properties account as principal characteristics for enhancing repair bond strength. Moreover, its application demonstrated in the present study, as well as in many others [9,11,18,19,21,47,48], to be a crucial step in the resin composite repair procedure.

The results of this study were not able to conclusively determine the best protocol for resin composite repair. Analysis of the diverse variables influencing the repair process has led to the conclusion that there is probably not one, but many different effective protocols to achieve a reliable repair. Despite this, some clinical recommendations can be drawn from the observed results. (I) As no significant differences were observed among mechanical surface treatments, utilization of more complex procedures like sandblasting can be avoided, especially if adjacent enamel is exposed [49]; (II) Considering the improved surface characteristics achieved with phosphoric acid, its application should be a routine step in repair procedures; (III) Utilization of an adhesive system is mandatory and does not involve an additional step, as the repair process often includes adhesion to both enamel and dentin [36].

## 4. Experimental Section

### 4.1. Specimen Fabrication and Aging Procedure

**Figure 2:**
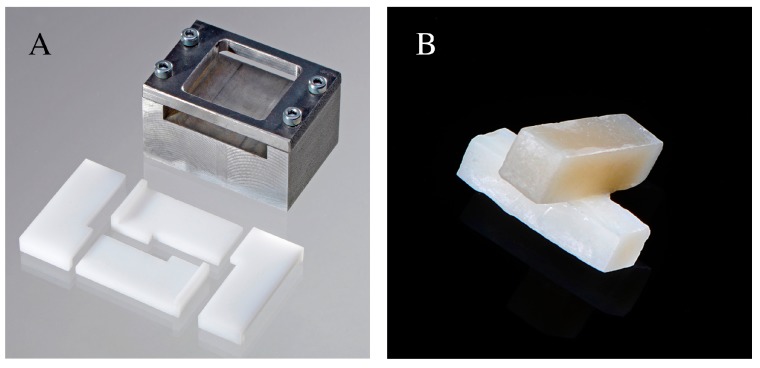
(**A**) Polyacetal Delrin molds used to produce the composite samples; (**B**) Bonded specimens.

One hundred and sixty-five bar specimens (3 × 3 × 10 mm^3^) were fabricated by inserting a nanohybrid resin composite (Grandio SO-Voco, Cuxhaven, Germany) shade A4 into two L-shaped Delrin polyacetal molds contained in a specific metallic holder (Figure 2A). An incremental technique (2 layers of 1.5 mm each) was used, in order to reduce polymerization shrinkage and to ensure proper polymerization of the material. A transparent mylar strip (Scheu-Dental, Iserlohn, Germany) was placed between the resin composite and the metallic holder to avoid contamination, as well as on top of the inserted material, to prevent formation of an oxygen inhibition layer. The molds were isolated with an insulating pen (Signum, Haereus-Kulzer, Hanau, Germany) to prevent resin composite bonding. A halogen light (EliparTrilight, 3M ESPE, St. Paul, MN, USA) with an output intensity of 800 mW/cm^2^ was applied at three overlapping spots (20 s each) for both incremental layers. After removal of the molds, subsequent post curing for 300 s was achieved in a light-oven (Unilux AC, Kulzer, Wehrheim, Germany). This was undertaken to reduce to a minimum the presence of unreacted groups in the resin composite. The specimens were then stored in distilled water at 37 °C for 30 days. Thereafter, one of their long sides was roughened with a red code diamond bur (grain size 27–76 µm), except for Group 3 (*n* = 15) where a blue code diamond bur (grain size 64–126 µm) was used. A material removal of 0.3 mm was achieved using a special device to ensure that grinding was performed parallel to the surface.

### 4.2. Surface Treatment and Resin Composite Bonding

The aged specimens were divided into eleven groups (*n* = 15) and assigned to different mechanical and/or chemical surface treatment protocols, as described in Table 1. Information about materials used and their composition is presented in Table 3.

The specimens were then repositioned in the L-shaped Delrin molds with the treated surface facing up. A second pair of molds was perpendicularly positioned so the new resin composite would form a cross with the aged bar (Figure 2B). Shade A1 Grandio SO resin composite was inserted into the molds and a mylar strip was placed on top of them. The same light curing protocol used for the A4 specimens was applied. Additionally, thirty new samples were fabricated following the same protocol described in Section 4.1, except for the absence of the mylar strip on top, which was carefully removed before light curing. In this manner, an oxygen inhibition layer was allowed to form. Immediately thereafter the A1 shade resin composite was inserted to form the previously described cross. Half of them (*n* = 15) were then stored in distilled water at 37 °C for 30 days, while the other half did not receive any further treatment and were tested within 24 h after fabrication. These additional specimens acted as positive reference groups.

### 4.3. Tensile Bond Strength (TBS)

A specific equiaxial loading setup proposed by Lohbauer et al. was used [50]. This test design ensures pure tensile loading, avoiding shear stresses, as well as a representative and well defined bonding area. The aged resin composite (A4) was fixed with a clamp-like holder while the extremes of the repair bar (A1) were put in tension by means of a thread and a load balancing roller grip [50]. A universal testing machine (Z 2.5; Zwick, Ulm, Germany) was used to measure the TBS, at a crosshead speed of 1 mm/min. The adhesive surface (about 9 mm^2^) was measured under a light stereomicroscope (SV 6, Zeiss, Jena, Germany) and then used to calculate the bond strength in MPa. The Kolmogorov-Smirnov test at α = 0.05 was applied to confirm the normal distribution of the results. A one-way analysis of variance (ANOVA) and the Student–Newman–Keuls test were used for pairwise comparisons (α = 0.05). Statistical analyses were performed with the SPSS Statistics 21.0 software (IBM, Chicago, IL, USA). Failure mode was analyzed under the light stereomicroscope at 50× magnification and classified as adhesive (at the composite–composite interface), cohesive (within one of the resin composite bars) or mixed.

### 4.4. Surface Characterization: SEM and Profilometric Evaluation

Six additional specimens of the Groups 1–4 and 6 were produced to observe the effect of their respective surface treatments. After ultrasonification, half of the treated surfaces in each group were gold sputtered and qualitatively evaluated under SEM (Leitz ISI-SR-50, Akashi, Japan). The other half was examined with a high resolution optical profilometer (CyberSCAN CT 100, Cyber Technologies, Ingolstadt, Germany) equipped with a white light sensor (vertical resolution of ±0.02 µm). Four different regions (1 × 1 mm^2^) of each specimen’s surface were measured to obtain the average surface roughness (Ra) and the mean roughness depth (Rz).

### 4.5. Degree of Conversion: FTIR-ATR Spectroscopy

To observe the effect of water aging on the resin composite degree of conversion, twenty new specimens were produced following the same procedure described in Section 4.1. Half of them were stored in water for 30 days, while the other half were fabricated 16 h before measurement. A material reduction of 0.3 mm was accomplished with SiC paper under water cooling at one of the surfaces in order to mimic the experimental conditions. After fine polishing (up to 4000 Grit), the specimens were subjected to FTIR spectroscopy (Impact 420, Nicolet Instruments, Madison, WI, USA) with attenuated total reflectance method (ATR, DuraSamplIR II, SensiIR Technologies, Danbury, CT, USA). The absorption spectrum of the samples, as well as of the uncured material (*n* = 10), was measured 64 times within a wave length spectrum of 4000 cm^−1^ to 650 cm^−1^. The peak heights for the aliphatic (C=C; at 1637 cm^−1^) and aromatic (C···C; at 1608 cm^−1^) carbon double bonds were recorded and used for the calculation of the degree of conversion (DC):

(1) $$\text{DC}=\left[1-\;\left\{\frac{\text{Abs }{\left(C=C\right)}_{\text{cured}}\times \text{Abs }{\left(C\cdot \cdot \cdot C\right)}_{\text{uncured}}}{\text{Abs }{\left(C=C\right)}_{\text{uncured}}\;\times \text{Abs }{\left(C\cdot \cdot \cdot C\right)}_{\text{cured}}}\right\}\right]\;\times 100$$

where Abs stands for absorption spectrum at the specified peaks for the cured and uncured material.

## 5. Conclusions

Within the limits of this study, the following conclusions can be drawn:

- A considerable decrease in the availability of unreacted carbon double bonds was observed after aging.
- The increase in TBS values was not directly correlated to the surface roughness profiles measured.
- Cleaning with phosphoric acid significantly improved TBS.
- The use of a bonding system resulted critical to achieve reliable bond strengths.

## Figures and Tables

**Table 1:** Surface treatments and tensile bond strength (TBS) results (MPa).

Group	Surface Treatment	TBS (SD)
Group 1 *Negative Reference*	Red code diamond bur (grain size 27–76 µm)	4.86 ^a^ (±1.06)
Group 2	Etching with 35% phosphoric acid (Scotchbond Etchant, 3M ESPE, St. Paul, MN, USA) for 15 s	6.75 ^b^ (±1.40)
Group 3	Blue code diamond bur (grain size 64–126 µm)	7.15 ^b^ (±1.85)
Group 4	Sandblasting (CoJet-System, 3M ESPE, Seefeld, Germany) with 35 µm Al_2_O_3_ particles (2.8 bar for 4 s at 20 mm distance)	7.9 ^b^ (±1.64)
Group 5	Silane application for 60 s	6.62 ^b^ (±1.59)
Group 6	Sandblasting with 30 µm CoJet Sand (2.8 bar for 4 s at 20 mm distance) and subsequent silane application for 60 s	7.75 ^b^ (±1.87)
Group 7	Application of Syntac Primer for 15 s and careful drying with compressed air	9.82 ^c,d^ (±1.76)
Group 8	Application of Syntac Adhesive for 10 s and careful drying with compressed air	10.03 ^c,d^ (±1.51)
Group 9	Application of Heliobond for 60 s, careful drying with compressed air and light polymerization for 40 s	9.35 ^c^ (±2.05)
Group 10	Application of Syntac Primer + Adhesive and subsequent application of Heliobond	9.67 ^c,d^ (±1.88)
Group 11	Etching with 35% phosphoric acid followed by Syntac Primer + Adhesive and Heliobond	11.33 ^d^ (±2.03)
*Positive reference*	No surface treatment, no aging after repair	10.07 ^c,d^ (±1.54)
*Positive reference*	No surface treatment. After repair, aged in distilled water for 30 days	10.54 ^c,d^ (±2.04)

Means followed by the same superscript letters are not statistically different (at *p* < 0.05).

**Table 2:** Average surface roughness (Ra) and mean roughness depth (Rz) for the different surface treatments.

Group	Ra (µm)	Rz (µm)
Group 1—Negative reference *Red code bur*	1.07 ± 0.05	3.69 ± 0.25
Group 2 *Phosphoric acid*	1.15 ± 0.21	3.92 ± 0.77
Group 3 *Blue code bur*	3.36 ± 0.51	10.36 ± 1.55
Group 4 *Al*_2_*O*_3_ *sandblasting*	0.73 ± 0.05	2.5 ± 0.18
Group 6 *CoJet Sand*	0.81 ± 0.07	2.81 ± 0.21

**Table 3:** Materials used (information supplied by the manufacturers).

Material	Composition
Grandio SO * Voco Cuxhaven, Germany	Filler (89 wt %): 0.5–3 µm glass ceramic particles 0–40 nm SiO_2_ nanoparticles Matrix: Bis-GMA, Bis-EMA, TEGDMA
CoJet Sand 3M ESPE Seefeld, Germany	30 µm Al_2_O_3_ silicatized particles
Monobond Plus Ivoclar Vivadent Schaan, Lichtenstein	3-trimethoxysilylpropyl methacrylate (<2.5%) Methacrylated phosphoric acid ester (<2.5%) Ethanol (50%–100%)
Syntac Ivoclar Vivadent Schaan, Lichtenstein	Primer: TEGDMA, PEGDMA (25%) Maleic acid (2.5%–10%) Acetone (25%–50%) Adhesive: PEGDMA (25%–50%) Glutaraldehyde (2.5%–10%) Water (60%)
Heliobond Ivoclar Vivadent Schaan, Lichtenstein	Bis-GMA (50%–60%) TEGDMA (25%–50%)

Bis-GMA: Bisphenol A diglycidylmethacrylate; Bis-EMA: Ethoxylated bisphenol A dimethacrylate; TEGDMA: triethylene glycol dimethacrylate; Al_2_O_3_: aluminium oxide; PEGDMA: polyethylene glycol dimethacrylate. * LOT shade A1: 1103472/12-13; LOT shade A4: 1221238/01-15.
